# Cytochrome b5 reductase 2 suppresses tumor formation in nasopharyngeal carcinoma by attenuating angiogenesis

**DOI:** 10.1186/s40880-015-0044-4

**Published:** 2015-08-15

**Authors:** Huixin Ming, Ying Lan, Feng He, Xue Xiao, Xiaoying Zhou, Zhe Zhang, Ping Li, Guangwu Huang

**Affiliations:** Department of Pathology, First Affiliated Hospital of Guangxi Medical University, Nanning, Guangxi 530021 P.R. China; Department of Otolaryngology-Head and Neck Surgery, First Affiliated Hospital of Guangxi Medical University, Nanning, Guangxi 530021 P.R. China; Medical Research Centre, Guangxi Medical University, Nanning, Guangxi 530021 P.R. China

**Keywords:** Cytochrome b5 reductase 2, Nasopharyngeal carcinoma, Chick embryo model, Angiogenesis

## Abstract

**Background:**

Cytochrome b5 reductase 2 (CYB5R2) is a potential tumor suppressor that inhibits cell proliferation and motility in nasopharyngeal carcinoma (NPC). Inactivation of CYB5R2 is associated with lymph node metastasis in NPC. This study aimed to explore the mechanisms contributing to the anti-neoplastic effects of CYB5R2.

**Methods:**

Polymerase chain reaction (PCR) assays were used to analyze the transcription of 84 genes known to be involved in representative cancer pathways in the NPC cell line HONE1. NPC cell lines CNE2 and HONE1 were transiently transfected with CYB5R2, and data was validated by real-time PCR. A chick chorioallantoic membrane (CAM) embryo model was implanted with CYB5R2-expressing CNE2 and HONE1 cells to evaluate the effect of CYB5R2 on angiogenesis. An immunohistochemical assay of the CAM model was used to analyze the protein expression of vascular endothelial growth factor (VEGF).

**Results:**

In CYB5R2-transfected NPC cells, PCR assays revealed up-regulated mRNA levels of Fas cell surface death receptor (*FAS*), FBJ murine osteosarcoma viral oncogene homolog (*FOS*), phosphoinositide-3-kinase regulatory subunit 1 (*PIK3R1*), integrin beta 3 (*ITGB3*), metastasis suppressor 1 (*MTSS1*), interferon beta 1 (*IFNB1*), and cyclin-dependent kinase inhibitor 2A (*CDKN2A*) and down-regulated levels of integrin beta 5 (*ITGB5*), insulin-like growth factor 1 (*IGF1*), TEK tyrosine kinase (*TEK*), transforming growth factor beta receptor 1 (*TGFBR1*), and *VEGF*. The angiogenesis in the CAM model implanted with CYB5R2-transfected NPC cells was inhibited. Down-regulation of VEGF by CYB5R2 in NPC cells was confirmed by immunohistochemical staining in the CAM model.

**Conclusion:**

CYB5R2 up-regulates the expression of genes that negatively modulate angiogenesis in NPC cells and down-regulates the expression of *VEGF* to reduce angiogenesis, thereby suppressing tumor formation.

## Background

Nasopharyngeal carcinoma (NPC) is a major health problem in South China, with a morbidity of 25–30 per 100,000 people each year [1]. The etiology of NPC is closely related to environmental carcinogens, Epstein-Barr virus (EBV) infection, and genetic susceptibility [2, 3]. Inactivation of tumor suppressor genes (TSGs) by promoter DNA hypermethylation is frequently observed in patients with NPC and is considered to be a tumorigenic factor of NPC [4].

Cytochrome b5 reductase 2 (CYB5R2), a nicotinamide adenine dinucleotide (NADH)-dependent flavin reductase, belongs to the cytochrome reductase family and has functions in oxidation reduction, drug metabolism, and methemoglobin reduction in erythrocytes [5]. A recent study has shown that CYB5R provides the electrons necessary for turnover of fatty acid desaturase in the desaturation process of fatty acids [6]. CYB5R may therefore be an important cofactor in lipid metabolism.

The expression of CYB5R2 varies in different types of cancer. CYB5R2 is abnormally inactivated in prostate and breast cancers, and it regulates cell proliferation and differentiation, similar to other TSGs [7, 8], but it is up-regulated in B cell acute lymphocytic leukemia [9].

In previous study, we showed that promoter DNA hypermethylation was responsible for inactivation of CYB5R2 and associated with lymph node metastasis in NPC patients [10]. In addition, exogenous expression of CYB5R2 significantly inhibited the proliferation, colony formation, migration, and in vivo tumor formation of NPC cells, which suggests that CYB5R2 is a potential TSG of NPC [10]. However, the molecular mechanism by which CYB5R2 acts as a TSG has not been illustrated.

In the present study, we further explored the pathways affected by CYB5R2. Using polymerase chain reaction (PCR) assays of NPC cells transfected with CYB5R2, we investigated the changes in mRNA expression of 84 genes in 6 signaling pathways related to cancer. A chick chorioallantoic membrane (CAM) embryo model was used to validate the inhibitory effect of CYB5R2 on angiogenesis in NPC cell xenograft.

## Methods

### Cell culture and reagents

EBV-negative NPC cell lines CNE2 [11] and HONE1 [12] were cultured in 6-well plates with 3 mL of Iscove’s modified Dulbecco’s medium (IMDM, Invitrogen, Carlsbad, CA, USA) containing 10% newborn calf serum (Gibco, Grand Island, NY, USA), 100 U/mL penicillin, and 100 μg/mL streptomycin in a humidified atmosphere with 5% CO_2_ at 37°C.

### PCR assay of cancer pathway genes

Total RNA was isolated from HONE1 cells, in which the transcription of CYB5R2 is almost silence, using Trizol (Invitrogen). First-strand cDNA was synthesized using M-MLV reverse transcriptase (Promega, Madison, WI, USA). The PAHS-033A Human Cancer Pathway Finder Superarray and Microsoft Excel-based data analysis software (Qiagen, Dusseldorf, Germany) were used to identify changes in the mRNA expression of 84 genes that are representative of 6 signaling pathways: cell cycle control and DNA damage repair, apoptosis and cell senescence, signal transduction molecules and transcription factors, cell adhesion, angiogenesis and tumor invasion, and metastasis.

### Vector construction and transfection

Full-length cDNA from the open reading frame of CYB5R2, purchased from Origen Co. (Beijing, China), was subcloned into the pCMV-Tag3A vector (Stratagene, La Jolla, CA, USA). CNE2 or HONE1 cells were seeded in 6-well plates (8 × 10^5^ cells/well) and transfected with 2 μg pCMV-Tag3A-CYB5R2 or pCMV-Tag3A (empty vector) plasmids using an X-treme GENE HP DNA Transfection Reagent (Roche Diagnostic, Penzberg, Germany) and incubated for 48 h. Each experiment was repeated three times.

### Quantitative real-time PCR

Significant modification of gene transcription by ectopic expression of CYB5R2 was further confirmed by quantitative real-time PCR. Primer information is in Table 1. GAPDH was amplified from the same cDNA samples and was used as an internal control. The PCR conditions were 95°C for 10 min, followed by 40 cycles at 95°C for 30 s and 60°C for 1 min. Relative expression of target genes in CNE2 and HONE1 cells transfected with pCMV-Tag3A-CYB5R2 was determined by the 2^−△△CT^ method as follows: Q = 2^−ΔΔCT^, ΔΔCT = (CT_target gene_ − CT_GAPDH_)_experimental_ − (CT_target gene_ − CT_GAPDH_)_control_. Empty vector-transfected cells were used as the control. Independent experiments were performed in triplicate.

**Table 1:** Primer sequences used in this study

Primer	Primer sequence (5′–3′)	Product size (bp)
CYB5R2	Forward: AAACACTGGCCGATCACCT	150
	Reverse: TGACCAAGATATCCTCCTCTGT	
FAS	Forward: CTGCCATAAGCCCTGTCCTCCA	199
	Reverse: ATTCTGGGTCCGGGTGCAGTT	
FOS	Forward: TGGCAGGAGGGGCAAGGTGGA	240
	Reverse: GCAGGTCGGTGAGCTGCCAGGATG	
PIK3R1	Forward: CGGCAAAAGAAGTTGAACGAGTGG	347
	Reverse: TGCACAAGGGAGGTGTGTTGGTAA	
ITGB3	Forward: GTAACCTGCGGATTGGCTTCG	170
	Reverse: GAAGCGGGTCACCTGGTCAGT	
MTSS1	Forward: GTGGTGGGACCAGGGAGATTG	232
	Reverse: TTCAGCGTATCCGAGGACTTCTT	
ITGB5	Forward: CCGGCTCGCAGGTCTCAAC	306
	Reverse: TCACCGGGCCGGAGGTTCAC	
IFNB1	Forward: GCTCTCCTGTTGTGCTTCTCCACT	176
	Reverse: AGCTGCTTAATCTCCTCAGGGATG	
VEGF	Forward: CTTCTGAGTTGCCCAGGAGACCACT	230
	Reverse: TCAACCACTCACACACACACAACCA	
IGF1	Forward: CAACAAGCCCACAGGGTATGGCT	173
	Reverse: TGGGCATGTCGGTGTGGCGCT	
TEK	Forward: CAGGAGTTTGGGTCTGCAGTGTGA	383
	Reverse: TGGAGGAGGGAGTCCGATAGAAGC	
TGFBR1	Forward: GCATTGGCAAAGGTCGATTTGG	303
	Reverse: TCGCCGTGGACAGAGCAAGTT	
CDKN2A	Forward: CGAAGGTCCTACAGGGCCACAAC	273
	Reverse: CTCGCAAGAAATGCCCACATGAA	
GAPDH	Forward: AAGCTCACTGGCATGGCCTT	375
	Reverse: CTCTCTTCCTCTTGTGCTCTTG	
HIF-1α	Forward: CCGAATTGATGGGATATGAG	150
	Reverse: TCATGATGAGTTTTGGTCAGATG	

*CYB5R2* cytochrome b5 reductase 2, *FAS* Fas cell surface death receptor, *FOS* FBJ murine osteosarcoma viral oncogene homolog, *PIK3R1* phosphoinositide-3-kinase regulatory subunit 1, *ITGB3* integrin beta 3, *MTSS1* metastasis suppressor 1, *ITGB5* integrin beta 5, *IFNB1* interferon beta 1, *VEGF* vascular endothelial growth factor, *IGF1* insulin-like growth factor 1, *TEK* TEK tyrosine kinase, *TGFBR1* transforming growth factor beta receptor 1, *CDKN2A* cyclin-dependent kinase inhibitor 2A, *GAPDH* glyceraldehyde-3-phosphate dehydrogenase, *HIF-1α* hypoxia-induced factor-1 alpha.

### CAM assay

Twelve fertilized chicken eggs were incubated for 8 days at 37°C at 60% humidity. Briefly, a small hole was drilled into the eggshell where the air sac is located. For inoculation, a 1-cm^2^ window was carefully opened. An amount of 8 × 10^5^ CNE2 or HONE1 cells was resuspended in 10 μL of IMDM and seeded into a silicon ring (φ = 5 mm, homemade), which was then placed on the CAM. The window was covered with parafilm (Honsmed, Shanghai, China), and the egg was placed back into the incubator. Silicone rings were removed 24 h later [13]. Tumor growth and vessels were observed daily using a SZ61 Zoom Stereo Microscope (Olympus, Tokyo, Japan). The area under the opened window and the vascular area were analyzed with Image-Pro Plus 5.0 (Media Cybernetics, Rockville, MD, USA). Angiogenesis was quantified as the ratio of vascular area to total observed area. Micro-tumors were removed from CAMs on day 5 after cell implantation, and the tumor volume was calculated as volume = width^2^ × length × 0.5 [14]. Tumors were fixed with 4% paraformaldehyde and embedded in paraffin.

### Hematoxylin and Eosin (H&E) staining and immunohistochemistry (IHC assay)

H&E staining was performed following a standard protocol [15]. For immunohistochemical staining, sections (3 μm) were deparaffinized and rehydrated. Sections then underwent antigen retrieval with 0.01 mol/L sodium citrate, followed by blocking with 5% fetal bovine serum and incubation with 50 μL of ready-for-use monoclonal antibody targeting vascular endothelial growth factor (VEGF) (MAB-0243, MAIXIN-BIO, Fuzhou, Fujian, China) at 4°C overnight. Sections were then exposed to 1:1,000 peroxidase-conjugated goat anti-mouse secondary antibody (ZB-2305, ZSGB-BIO, Beijing, China) at room temperature for 30 min, and signals were visualized using 3,3′-diaminobenzidine (DAB) reagent (ZSGB-BIO). Finally, sections were counterstained with hematoxylin and images were acquired under a C-5050 Olympus microscope (Olympus).

### Enzyme-linked immunosorbent assay (ELISA)

ELISA of secreted VEGF was carried out following the manufacturer’s instructions (Sigma, Louis, MO, USA). Standard and cell culture supernatant (100 μL/well) was added to 96-well plates, and cells were incubated for 3 h at room temperature followed by coloration with horseradish peroxidase-conjugated streptavidin and 5′-tetramethylbenzidinev (TMB) reagent. Absorbance was measured using a microplate reader (Multiskan Fisher Scientific, Thermo, Waltham, MA, USA) at a wavelength of 450 nm. All samples were tested in triplicate.

### Statistical analysis

Statistical analysis involved the use of SPSS version 16.0 (SPSS Inc., Chicago, IL, USA). Data are shown as the mean ± standard deviation (SD) of 3 experiments. An independent sample *t* test was used to compare data. *P* < 0.05 was considered statistically significant.

## Results

### CYB5R2 affected the expression of genes associated with apoptosis, cell cycle, angiogenesis, invasion, and metastasis of NPC cells

**Figure 1:**
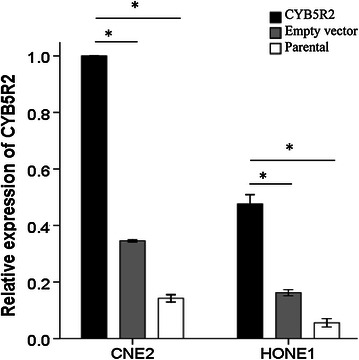
Ectopic expression of cytochrome b5 reductase 2 (CYB5R2) in nasopharyngeal carcinoma (NPC) cell lines CNE2 and HONE1. Relative expression of CYB5R2 in NPC cell lines was confirmed by real-time polymerase chain reaction (PCR) 48 h after transfection of CYB5R2 or empty vector plasmids. Data are presented as mean ± standard deviation (SD) of three experiments. *P < 0.05.

**Figure 2:**
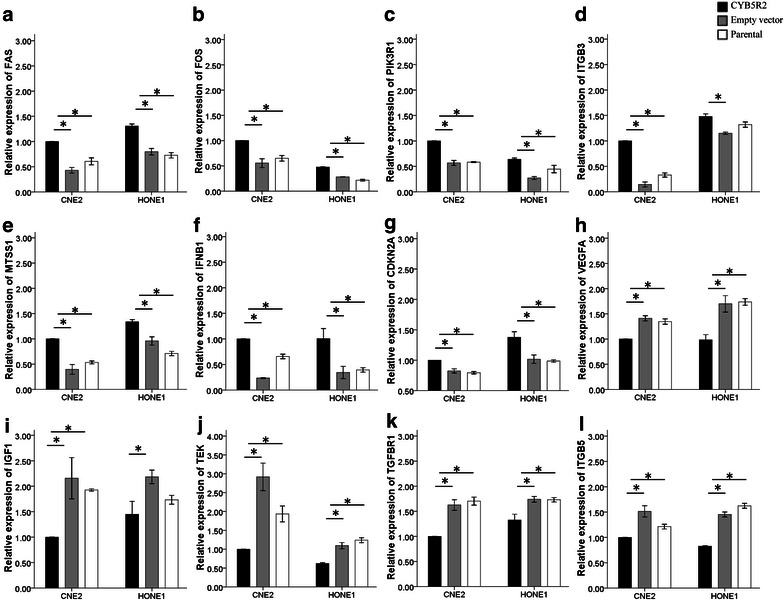
Real-time PCR confirmation of genes regulated by CYB5R2 in NPC cell lines CNE2 and HONE1. **a** Fas cell surface death receptor (FAS); **b** FBJ murine osteosarcoma viral oncogene homolog (FOS); **c** phosphoinositide-3-kinase regulatory subunit 1 (PIK3R1); **d** integrin beta 3 (ITGB3); **e** metastasis suppressor 1 (MTSS1); **f** interferon beta 1 (IFNB1); **g** cyclin-dependent kinase inhibitor 2A (CDKN2A); **h** vascular endothelial growth factor A (VEGFA); **i** insulin-like growth factor 1 (IGF1); **j** TEK tyrosine kinase (TEK); **k** transforming growth factor beta receptor 1 (TGFBR1); **l** integrin beta (ITGB5). Data are presented as mean ± SD of three experiments. *P < 0.05.

To determine the mechanisms involved in the tumor suppressive effect of CYB5R2 on NPC cells, we first confirmed that CYB5R2 mRNA level was increased in NPC cells (CNE2 and HONE1) after transfection with a CYB5R2 expression plasmid (Figure 1). We then used PCR assays to evaluate changes in the mRNA levels of 84 genes related to cell proliferation, apoptosis, cell cycle, angiogenesis, invasion, and metastasis in HONE1 cells that were transfected with CYB5R2 or an empty vector. The mRNA levels of 12 genes were significantly altered by CYB5R2 overexpression (up- or down-regulated by at least 1.5-fold compared with empty vector control) (Table 2). Several genes involved in apoptosis, signal transduction and transcription, cell cycle control, and DNA damage repair [Fas cell surface death receptor (*FAS*), FBJ murine osteosarcoma viral oncogene homolog (*FOS*), phosphoinositide-3-kinase regulatory subunit 1 (*PIK3R1*), and cyclin-dependent kinase inhibitor 2A (*CDKN2A*)] were up-regulated. Some genes involved in angiogenesis (vascular endothelial growth factor A (*VEGFA*) [16]), insulin-like growth factor 1 (*IGF1*), TEK tyrosine kinase (*TEK*), and transforming growth factor beta receptor 1 (*TGFBR1*) were down-regulated, whereas one, interferon beta 1 (*IFNB1*), was up-regulated. The mRNA levels of several genes involved in adhesion [integrin beta 3 (*ITGB3*), metastasis suppressor 1 (*MTSS1*), and integrin beta 5 (*ITGB5*)] were up-regulated or down-regulated with CYB5R2 expression. These changes in the gene expression in CYB5R2-transfected NPC cells (CNE2 and HONE1) was verified by real-time PCR (Figure 2).

**Table 2:** Genes up-regulated or down-regulated at least 1.5-fold by overexpression of CYB5R2 in HONE1 cells

Pathway	Gene symbol	Name of gene	Fold change in expression
Apoptosis and cell senescence	*FAS*	Fas (tumor necrosis factor receptor superfamily member 6)	1.81
Signal transduction and transcription	*FOS*	FBJ murine osteosarcoma viral oncogene homolog	1.86
	*PIK3R1*	Phosphoinositide-3-kinase regulatory subunit 1 (alpha)	1.57
Adhesion	*ITGB3*	Integrin beta 3 (platelet glycoprotein IIIa, antigen CD61)	1.68
	*MTSS1*	Metastasis suppressor 1	1.59
	*ITGB5*	Integrin beta 5	−8.72
Angiogenesis	*IFNB1*	Interferon beta 1, fibroblast	1.84
	*VEGFA*	Vascular endothelial growth factor A	−1.69
	*IGF1*	Insulin-like growth factor 1 (somatomedin C)	−1.57
	*TEK*	TEK tyrosine kinase, endothelial	−10.28
	*TGFBR1*	Transforming growth factor beta receptor 1	−1.71
Cell cycle control and DNA damage repair	*CDKN2A*	Cyclin-dependent kinase inhibitor 2A	1.57

### Ectopic expression of CYB5R2 suppressed angiogenic capacity in NPC cells

**Figure 3:**
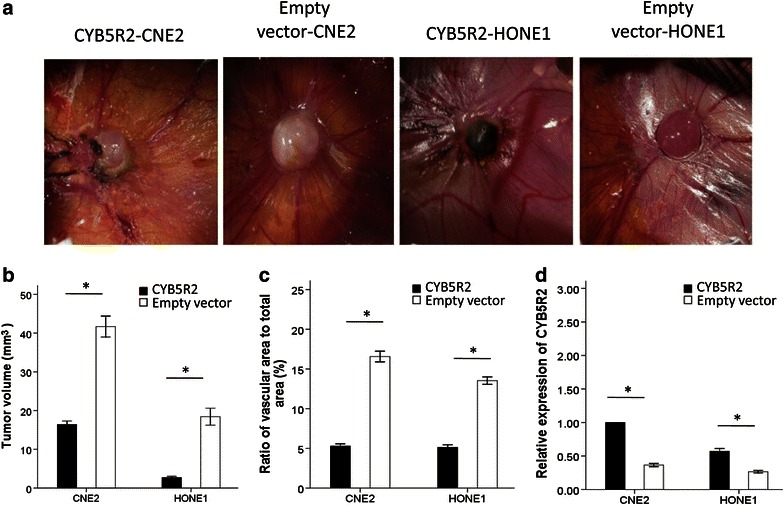
CYB5R2 inhibits angiogenesis in NPC cell xenografts in a chick chorioallantoic membrane (CAM) model. **a** Representative images of xenograft tumors and surrounding blood vessels at 5 days after implantation with CNE2 or HONE1 cells that were transfected with CYB5R2 expression plasmid or empty vector plasmid. **b** Tumor volume measured on images of **a**. **c** Blood vessel area measured on images of **a**. **d** Relative expression of CYB5R2 in tumors from CAM models at 5 days after implantation. Data are presented as mean ± SD of three experiments. *P < 0.05.

To evaluate the influence of exogenously expressed CYB5R2 on the angiogenic capacity of NPC cells, we implanted CYB5R2-transfected CNE2 and HONE1 cells into a CAM model to analyze the formation of tumors and angiogenesis. CYB5R2-transfected CNE2 and HONE1 cells formed smaller tumors than empty vector- cells (Figure 3a, b), and this was consistent with our previous in vivo results in nude mice [10].

Empty vector-transfected CNE2 and HONE1 cells showed dramatically increased blood vessel formation around the tumor mass, whereas the density of vessels was decreased with CYB5R2 overexpression (Figure 3a, c). Neovascularization was calculated as 5.3% for CYB5R2-transfected CNE2 cells and 5.1% for CYB5R2-transfected HONE1 cells, which were significantly less than those for empty vector-transfected CNE2 and HONE1 cells (16.6% and 13.5%, respectively, both *P* < 0.001; Figure 3c). The mRNA level of *CYB5R2* was higher in the xenografts derived from CYB5R2-transfected CNE2 and HONE1 cells than from empty vector-transfected cells (Figure 3d).

**Figure 4:**
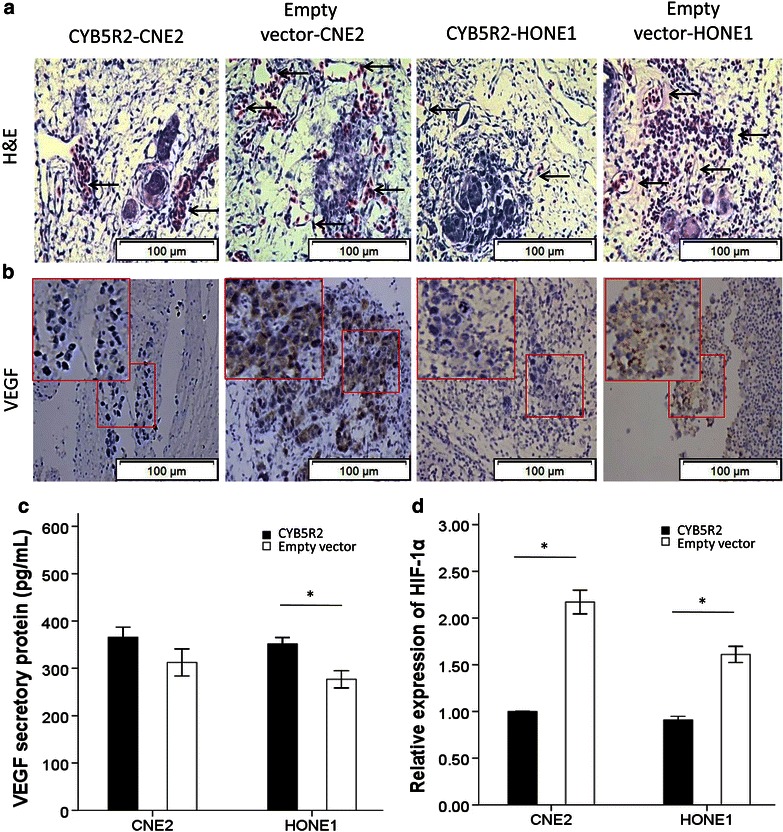
Overexpression of CYB5R2 in NPC cells inhibits angiogenesis and VEGF expression. **a** Hematoxylin and Eosin (H&E) staining of xenografts in paraffin-embedded sections with tumors from the CAM model*. Black arrows* point to microvessels around the tumor. **b** Immunohistochemical staining of VEGF in xenografts from the CAM model. Empty vector-transfected CNE2 and HONE1 cells are positive for VEGF. **c** Enzyme-linked immunosorbent assay (ELISA) of secreted VEGF protein in supernatant from CYB5R2- or empty vector-transfected CNE2 and HONE1 cells. **d** Real-time PCR analysis of the relative mRNA level of hypoxia-induced factor alpha (HIF-1α) in CYB5R2- or empty vector-transfected CNE2 and HONE1 cells. Data are presented as mean ± SD of three experiments. *P < 0.05.

H&E staining revealed that the induction of microvessels with CYB5R2-transfected CNE2 and HONE1 cell implantation in CAM was reduced compared with that empty vector-transfected cell implantation (Figure 4a). The protein level of VEGF was decreased in CYB5R2-expressing CNE2 and HONE1 cells, verifying the inhibitory effect of CYB5R2 on angiogenesis (Figure 4b). Secreted VEGF protein was also reduced by overexpression of CYB5R2 in both CNE2 and HONE1 cells (Figure 4c). Finally, hypoxia-induced factor alpha (HIF-1α), an important activator of VEGF expression, was suppressed by CYB5R2 overexpression (Figure 4d), which may explain the inhibitory effect of CYB5R2 on VEGF expression.

## Discussion

We found, using PCR assays, that the overexpression of *CYB5R2*, a potential TSG, affected the transcription of genes involved in several major cancer-related pathways, including apoptosis, cell cycle, cell adhesion, angiogenesis, and tumor invasion and metastasis. Five genes (*VEGFA*, *IFNB1*, *IGF1*, *TEK*, and *TGFBR1*) are associated with angiogenesis, which provides clues to the mechanism of the association between epigenetic inactivation of *CYB5R2* and lymph node metastasis [10]. We focused on the effect of CYB5R2 on the formation of new vasculature in NPC. Ectopic expression of CYB5R2, by NPC cells implanted in a CAM model, inhibited angiogenesis. Down-regulation of VEGF by CYB5R2 in NPC cells was confirmed by immunohistochemical staining in the CAM model. CYB5R2 overexpression in NPC cells up-regulated the expression of genes that negatively modulate angiogenesis and down-regulated the expression of the pro-angiogenic factor VEGF, resulting in reduced angiogenesis and, thereby, a suppression of tumor formation.

The presence of palpable cervical lymph nodes is usually the first presenting symptom in NPC, indicating that NPC has a high propensity to invade the lymphatic system and spread to the cervical lymph nodes. NPC cells may also spread to distant organs, including the lungs, liver, and bones, through the blood stream. Angiogenesis and lymphangiogenesis are essential for tumor growth and metastasis, which is primarily driven by VEGF. As a member of the platelet-derived growth factor family, VEGF is the most influential stimulator of vascular endothelial growth during tumor angiogenesis [17]. VEGF is now considered to be the most potent angiogenic factor and, therefore, a promising therapeutic target for treating cancer [18, 19]. VEGF is highly expressed in a variety of human tumors and is closely related to a poor outcome in patients with NPC [20, 21]. VEGF is regulated by HIF-1α [22] and by oncogene signaling factors including platelet-derived growth factor, fibroblast growth factor, epidermal growth factor (EGF), tumor necrosis factor, and transforming growth factor-β [23]. VEGF binds primarily to VEGF receptor type 2 in endothelial cells, by which it stimulates angiogenesis by activating downstream signaling enzymes, including extracellular signal-regulated kinase 1/2, Akt, and endothelial nitric oxide synthase [24]. IFNB1 is an ISGylation modification inducer [25] that inhibits tumor angiogenesis by repressing pro-angiogenic genes, including VEGF and matrix metalloproteinase 9 [26]. IGF1 and TGFBR1 play important roles in inducing angiogenesis by increasing the level of VEGF [27–29]. TEK encodes a receptor that has 2 Ig-like domains, 3 EGF-like domains, and 3 fibronectin type III repeats and functions in inducing blood vessel growth and affecting the establishment, growth, and metastasis of tumors through activation of the Akt signaling pathway [30, 31].

We found that CYB5R2 expression in NPC cells altered the angiogenic balance by down-regulating a series of angiogenesis-promoting factors (VEGFA, IGF1, TGFBR1, and TEK) and up-regulating an angiogenesis inhibitor, IFNB1. These data imply that CYB5R2 plays a role in angiogenic suppression. To further investigate this role, we used CAM, an in vivo model, to study tumor growth and angiogenesis and to evaluate the effect of CYB5R2 on angiogenesis [32, 33]. CYB5R2-transfected CNE2 and HONE1 cell xenografts were smaller than empty vector-transfected CNE2 and HONE1 cell xenografts. Angiogenesis on the surface of the growing CAM was significantly suppressed in CYB5R2-expressing NPC cell xenografts. VEGF protein levels in xenografts and secreted VEGF protein levels from NPC cells were both reduced by CYB5R2 overexpression. This provides further support for a role of CYB5R2 in the down-regulation of VEGF mRNA levels and subsequent reduction in angiogenesis. The transcription of HIF-1α was also significantly down-regulated by CYB5R2 in NPC cells, which may further explain the down-regulation of VEGF by CYB5R2. CYB5R2 may therefore suppress tumor angiogenesis, but the detailed molecular mechanism has not yet been fully explained.

In summary, CYB5R2 transfection in NPC cells affected mRNA levels of several factors involved in angiogenesis, including VEGF, IFNB1, IGF1, TEK, and TGFBR1. When we overexpressed CYB5R2 in NPC cells and implanted these cells into a CAM model, cell proliferation and angiogenesis were suppressed. The malignant phenotype may be inhibited by CYB5R2 through a down-regulation of VEGF or suppression of HIF-1α expression. Inactivation of CYB5R2 may contribute to angiogenesis in NPC. The results of this study provide insight into the mechanism by which CYB5R2 acts to suppress tumor function, and further support CYB5R2 as a novel TSG in NPC.
